# A feasibility study with process evaluation of a preschool intervention to improve child and family lifestyle behaviours

**DOI:** 10.1186/s12889-017-4167-1

**Published:** 2017-03-11

**Authors:** Lorraine McSweeney, Vera Araújo-Soares, Tim Rapley, Ashley Adamson

**Affiliations:** 1 Institute of Health and Society and Human Nutrition Research Centre, Newcastle University, Framlington Place, Newcastle, NE2 4HH UK; 2 Institute of Health & Society, Newcastle University, Richardson Road, Newcastle, NE2 4AX UK

**Keywords:** Preschool, Family, Obesity, Behaviour change, Feasibility study

## Abstract

**Background:**

Around a fifth of children starting school in England are now overweight/obese. There is a paucity of interventions with the aim of obesity prevention in preschool-age children in the UK. Previous research has demonstrated some positive results in changing specific health behaviours, however, positive trends in overall obesity rates are lacking. Preschool settings may provide valuable opportunities to access children and their families not only for promoting healthy lifestyles, but also to develop and evaluate behaviour-change interventions.

**Methods:**

This paper presents a cluster randomised feasibility study of a theory based behaviour-change preschool practitioner-led intervention tested in four preschool centres in the North East of England. The primary outcome measures were to test the acceptability and feasibility of the data collection measures and intervention. Secondary measures were collected and reported for extra information. At baseline and post intervention, children’s anthropometric, dietary and physical activity measures as well as family ‘active’ time data were collected. The preschool practitioner-led intervention included family intervention tasks such as ‘family goal-setting activities’ and ‘cooking challenges’. Preschool activities included increasing physical activity and providing activities with the potential to change behaviour with increased knowledge of and acceptance of healthy eating. The process evaluation was an on-going monthly process and was collected in multiple forms such as questionnaires, photographs and verbal feedback.

**Results:**

‘Gatekeeper’ permission and lower-hierarchal adherence were initially a problem for recruitment and methods acceptance. However, at intervention end the preschool teachers and parents stated they found most intervention methods and activities acceptable, and some positive changes in family health behaviours were reported. However, the preschool centres appeared to have difficulties with enforcing everyday school healthy eating policies.

**Conclusions:**

The findings from the current study may have implications for nursery practitioners, nursery settings, Local Educational Authorities and policy makers, and contributes to the body of literature. However, further work with preschool practitioners is required to determine how personal attitudes and school policy application can be supported to implement successfully such an intervention.

**Trial registration:**

ISRCTN12345678 (16/02/17) retrospectively registered.

## Background

Around a fifth of children are starting school overweight in the United Kingdom [1]. Globally, in 2010 the number of overweight children under the age of five, was estimated to be over 42 million [2] Overweight 2- to 5 year-olds are at least four times more likely to become overweight adults [3] and disadvantaged members of the population may be more vulnerable to societal effects which may lead to obesity [4]. The development of childhood obesity is multifaceted as demonstrated by the Foresight obesity segmented map for children [5]. Long-term health risks of overweight and obesity include: obesity persistence; cardiovascular risk factors; stroke; gall bladder disease; diabetes; fatty liver disease; and some cancers [6–8]. It is proposed that ‘early childhood provides a unique opportunity within which to establish lifestyle behaviours that will promote health and minimise the risk of development of fatness’ ([9], p. 328). A number of influential independent reviews commissioned by the UK government have highlighted the importance of early intervention and support for families with young children [10–14]. As part of a 2007 UK government strategy in an attempt to reverse the obesity trend, a Public Service Agreement (PSA) aiming to improve the health and wellbeing of children and young people was established [15]. This aims to reduce the percentage of obese and overweight children to the year 2000 levels by 2020 [16].

The UK has become the highest spender on early years services (preschool – before formal education) in Europe [17]. In 2015 94% of 3-year olds and 99% of 4-year olds benefited from some form of free early education [18] at government maintained schools or in the private, voluntary or independent sector [19]. It follows then, that preschool settings may provide valuable opportunities to access children and their families not only for promoting healthy lifestyles, but also to develop and evaluate behaviour-change interventions [20, 21]. The World Health Organisation (WHO) Commission on Ending Childhood Obesity reported that progress in tackling childhood obesity had been slow and inconsistent and the preschool years have been highlighted as a critical period for obesity prevention [22].

Early years providers in England follow the mandatory ‘Framework for the Early Years Foundation Stage’ (EYFS) [23]. One component of which is ‘Physical development’, in which ‘Children must be helped to understand the importance of physical activity, and to make healthy choices in relation to food’ ([24] p. 8). Therefore, an intervention with the aim of preventing obesity and improving family lifestyle choices which builds on these guidelines should be within the remit of a preschool setting [25]. However, curriculum-based interventions rely on the motivation, training of staff and on their ability to deliver the programme [26].

There is a paucity of interventions targeting children in preschool settings in the UK and component descriptions are limited. Reviews of interventions to prevent obesity in preschool children suggest that behaviours thought to contribute to obesity (such as sedentary behaviour) may be positively influenced in preschool settings and that there was great scope for improving physical activity [27, 28]. One such study [29] demonstrated increased structured PA in the daily preschool curriculum and an improvement in the children’s motor skills by incorporating daily 10 min structured practitioner-led activities. Furthermore, parents’ ability to act upon their children’s developing food choices/preferences and attitudes, through their own modelling behaviours is a powerful educational tool [30]. The main social influences (subjective norms) on physical activity and eating are reported to be parents and teachers [31–33]. This belief is echoed in a review conducted by Skouteris et al. [34] which looked at parental influence in preschool interventions, they concluded that there was a need for interventions which target strategies and techniques to aid parents in modifying their child’s diet and physical activity patterns [34]. In addition, further ‘practice-based’ evidence is required [35]. However, several methodological limitations in reviewed studies have been reported such as lack of theoretical frameworks and objective behavioural measures [34, 36]. It is important to identify appropriate behaviour change techniques in an intervention as without standardised definitions of the techniques, it would be difficult to replicate effective interventions and to identify techniques contributing to effectiveness across interventions [37]. Moreover, it has been reported that conducting research in school environments can be challenging, with complex multi access issues that need to be addressed [38, 39]. Further research which is sensitive to the needs of preschool settings’ and parents’ environmental mechanisms is required to determine which factors and methods are most effective [36].

The Study of Kids in Preschool (SKIP) was a feasibility [40–42] cluster randomised study of a health behaviour-change, preschool teacher/practitioner-led intervention focusing on diet, physical activity and sedentary behaviours. The aim was to determine which methods, tasks and activities were acceptable to preschool teachers, practitioners and families with children aged 3–4 years. In the UK, preschool or nursery practitioners (NP) are also known as nursery nurses, nursery workers or early year’s educators. To avoid confusion, from this point they will be referred as NPs unless specifically referring to preschool teachers who have an additional teaching qualification and usually undertake a lead role in the centre. This paper presents a cluster randomised feasibility study of a theory based behaviour-change preschool practitioner-led intervention conducted in four preschool centres in the North East of England.

## Methods

### Design

**Fig. 1:**
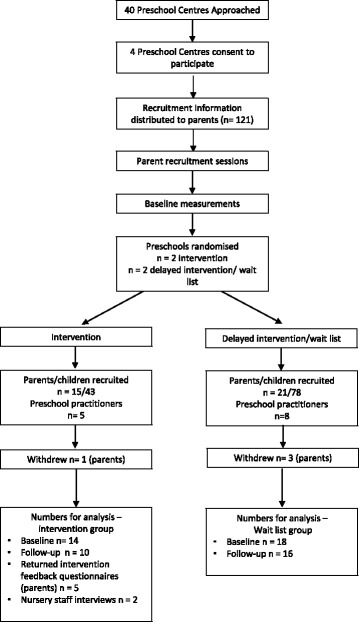
Flow diagram of the recruitment and analysis process

A cluster randomised feasibility trial. Four local government preschool centres associated with primary schools in the North East of England consented to participate and were randomised to either intervention or wait list control. See Fig. 1 for the Consort flow diagram which illustrates the numbers recruited, randomised, lost to follow-up and numbers for analysis. Two preschool centres received the intervention and the two control schools were placed on the wait list. Delivering a refined, delayed intervention provides an incentive for the wait list control preschools to participate, because instead of just providing control data, they later receive a refined intervention [43]. All four preschools were located in 10% of the most deprived locations of the region. In order to reduce selection bias the preschools were allocated to intervention or wait list control by a computerised programme by the Newcastle University Clinical Trials Unit.

### Participants

The main focus for this study was preschool children aged 3–5 years which local government preschools (nursery schools or nursery classes – henceforth reported as preschools) specifically cater for. It has been reported that families from a disadvantaged background are more likely to access preschool provision in these types of settings [44]. Therefore, the key inclusion criteria was any family with a preschool child (3–5 years) who attended a local government preschool centre (as described above).

#### Preschools

A convenience sampling strategy was employed to recruit preschools to the intervention. Forty preschools associated with primary schools were contacted by letter with a follow-up telephone call; head-teachers in four preschools consented to participate.

#### Families

Information letters were disseminated to all parents (*n* = 121) via the four preschool centres by staff. It was felt that face-to-face recruitment may prove effective and would give parents the opportunity to ask questions. Therefore ‘recruitment days’ were organised for each school. The researcher arranged to be present at the preschool when parents dropped off or collected their child; this was to provide further explanation about the study and to invite parents to participate.

### Intervention development

A primary qualitative study [45] which was conducted prior to this study, questioned parents and preschool centre staff of their views and knowledge of healthy eating promotion in preschool settings and informed the main goals and aims of the intervention. It was concluded that family friendly healthy eating strategies and activities should be developed and delivered in preschool settings in a manner that is sensitive to parents’ needs [45]. Preschool staff shared ideas and strategies for engaging parents and children.

The main goals of the intervention were to increase physical activity and healthy eating behaviours. The specific aims were distinct but complementary for the two contexts involved: preschool and family. For the preschool setting the specific aim was to increase physical activity. For the family setting the aims were to reduce TV viewing and increase family ‘active time’. For both settings the aims were to: a) reduce the consumption of high energy dense snacks, and; b) increase the consumption and the awareness of the importance of a ‘healthy’ breakfast.

#### Theoretical background

Two behaviour change models which were identified as good frameworks for intervention development were: The Social Cognitive Theory (SCT) [46] and Operant Conditioning (OC) [47]. SCT acknowledges the role of ‘modelling’ and contextual ‘availability’ as key in influencing a child’s dietary intake [47, 48]; especially in younger children who are potentially less influenced by peer pressure and more influenced by parent and teacher behaviours [31, 32, 49]. ‘Goal setting’ has demonstrated some success in adults [47] and helps people set their goals in motion in accordance with values, priorities and commitment to change [50]. Using behaviour change techniques such as ‘prompt specific goal setting’ [37] was considered to be an appropriate method to support parental behaviour change. OC theory and Social Learning Theory [48] uses reinforcement as one of the means of changing behaviour. Given that we are aiming at families with preschool-aged children, OC model seems suitable to support parents and NP in implementing changes with children [47]. In order to promote children’s engagement with healthy eating and physical activity, positive reinforcement strategies were used [51].

Table 1 describes the behaviour change techniques (BCT) used in this intervention, based on the CALO-RE Taxonomy of behaviour change techniques [52] as well as the targeted theoretical constructs (e.g., self-efficacy), implementation procedures (e.g., model/demonstrate behaviours), materials (e.g., parent information sheets) and intervention providers (e.g., preschool staff members). It is important to use specific BCTs that target hypothesised change mechanisms (based on the aforementioned theories) and that target in this case, both individual as well as environmental mechanisms.

**Table 1:** Behaviour change techniques, target variables, intervention procedures materials and providers

Behaviour change technique	Target Variable (IP: individual predictors; EF: environmental factors)	Procedures, Materials and Providers
Goal setting	IP: Outcome expectations, goals, self-efficacy EF: Environmental cues, barriers, access	Formulation of specific plans of how to reduce unhealthy snacking; increase healthy breakfast consumption, reduce TV viewing and increase family active time. Led by parents or staff. Sessions 2–5
Prompt self-monitoring of behaviour	IP: facilitate goal attainment, outcome expectations, self-efficacy	After an introductory session with the staff, parents were asked to keep a record of their behaviour related to goals set for behaviour change. Sessions 1–6
Model/demonstrate behaviour	IP: Skills, self-efficacy, expectations and beliefs EF: Facilitate vicarious learning	Parents were asked to model/demonstrate ‘healthy’ behaviours, being active and less sedentary. Staff were asked to model/demonstrate to children, ‘healthy’ eating and physical activity behaviours. The preschools received skipping ropes and the families received Frisbees. Sessions 3–6
Prompt practice	IP: Skills and knowledge EF: Environmental cues, access	Staff were asked to encourage parents to rehearse and repeat behaviours around set goals. Parents received activity and information sheets with suggestions for healthy eating and physical activity to facilitate building new habits/routines at home. Sessions 3–6
Provide rewards contingent on successful behaviour Social support	IP: Self-efficacy, Outcome expectations EF: Environmental cues	Staff were asked to praise and encourage positive behaviour changes in parents and children. Parents were asked to encourage to praise their children. Children received periodic prizes contingent on participating in healthy eating behaviour and physical activity. Families were given reward charts to use at home The preschools celebrated success with parties/fun days. Sessions 1–6
Provide instruction on how to perform the behaviour Social support	IP: Knowledge, skills EF: environmental cues	Parents received instruction from staff and via information sheets (see Table 2) on how to engage in healthy eating and physical activity behaviour. Children received instruction from staff in relation to healthy eating and practiced active skills such as jumping, skipping etc. Sessions 1–6
Provide information Social support	IP: Knowledge, skills EF: Environmental cues, access	Parents received general information about the programme and more detailed nutritional and physical activity information in the form of leaflets and activity sheets. Children received information from staff appropriate for their level of understanding. Sessions 1–6
Set graded tasks	IP Self efficacy, outcome expectations EF: Barriers, access	Within the goal setting sessions, staff were asked to encourage parents to set small achievable behaviour change goals (SMART) building on past successes. Staff were asked to encourage children to build on their past successes through play and activities. Sessions 1–6
Provide information about others’ approval/behaviour Social support	IP: Self-efficacy, outcome expectations EF: Environmental cues	Parents were encouraged to set up a peer group to support each other throughout the intervention. The peer group aimed to forge social support and act as models of the behaviour. Parents were invited to share ideas/successes on specially designated intervention notice board. Children were encouraged to display pictures and photos of their families’ activities and behaviours. Children were encouraged by staff reinforce peers’ behaviours/achievements. Sessions 1–6
Prompt barrier identification Coping/planning	IP: Self-efficacy, outcome expectations EF: Environmental cues, barriers, access	In their monthly meeting with staff; parents were encouraged to think about personal potential barriers to behaviour change and to identify ways of overcoming them. Session 2
Relapse prevention/coping planning	IP: Self –efficacy, goals	Based on identification of barriers; parents and staff were encouraged to work together to suggest strategies to overcome barriers. Session 5

#### Intervention components and delivery

Taking the family on board as a part of the intervention was integral to the generalisability and sustainability of the effects of the intervention. The ultimate target of the proposed intervention was the child whilst acknowledging that in order to reach the child, the intervention would need to act via preschool staff and parents [31, 32].

The intervention followed a detailed manual.^1^ Alongside the manual, preschool staff were invited to attend a 1 h intervention training session. This was led by the main author. The staff members were led through a series of interactive scenarios relating to: a) parenting styles – staff were shown photographs of parents demonstrating different types of parenting styles and were asked to discuss best practice and encouragement of optimum styles, b) working with parents – how to interact with parents using the Family Partnership Model, c) behaviour change techniques – how to encourage parents to set and monitor goals and, d) implementation of the intervention modules – how the modules could be implemented in their own setting. During the 5 months of the intervention period the main researcher visited each preschool centre once a month for approximately 30–60 min to discuss intervention procedures and techniques with preschool staff in preparation for each module.

The intervention titled: ‘Study of Kids in Preschool’ (SKIP) consisted of five monthly modules (see Table 2) plus an introductory session for parents. The five monthly preschool-based sessions targeted the child and aimed at increasing physical activity, knowledge of and prompting of healthy eating. Active BCTs delivered by the preschool staff provided information on modelling and demonstrating behaviour, prompting practice and providing instruction on how to perform the behaviour.

**Table 2:** Monthly intervention activities for preschool staff, parents and children

Month	Module title	Activities led by NPs	Activities led by and for parents	Activities for children
1	Introductory session	Meet with parents (reflection and goal-setting – group or individual) Activities with children	Analysing self-monitoring diary and goal setting (consented parents) Information sheets – ‘Activity Tips and Eating Well for 3–5 year olds’	Fruit tasting sessions Learning a new song – ‘Apples and Bananas’ Healthy meals and physical activity with parents
2	Five-a-day and Portion sizes	Monthly parent meetings Activities with children Update notice board	Reflection and monitoring of goals (consented parents) Information sheets- Snack tips, Five-a-day tips, portion size advice, snack and breakfast ideas, activity tips. Monthly challenges – The Carrot Challenge, No TV day, Our family achievement sheet, Our family reward chart	Rope and playground games Make ‘No TV’ signs Think of activities to do instead of watching TV Snack and art activities with carrots The Carrot challenge cooking activity with parents Physical activities with parents Learning a new song – ‘The Good Food Song’
3	Active Play	Monthly parent meetings Activities with children Update notice board	Reflection and monitoring of goals (consented parents) Information sheets – Meal times and drink facts, Snack and breakfast ideas, Active play Monthly challenges – The Apple Challenge, NO TV for 2 days, Family active challenge, Our family achievement sheet, Our family reward chart	Playground games and rope skills Learning about the importance of breakfast No TV signs and alternative activity ideas The Apple Challenge cooking activity with parents Physical activities with parents Activities with apples Learning a new song – ‘Round the Apple Tree’
4	Vegetable Tasters	Monthly parent meetings Activities with children Update notice board	Reflection and monitoring of goals (consented parents) Relapse prevention and coping strategies Information sheets – My child doesn’t like vegetables, Snack and breakfast ideas, Rainy day activities Monthly challenges – The Broccoli Challenge, No TV for 3 days, Family active challenge, Our family achievement sheet, Our family reward chart	Family activity ideas No TV ideas Activities with broccoli and vegetable tasting sessions The Broccoli Challenge cooking activity with parents Physical activities with parents Learning a new song – ‘Oh do you eat your vegetables?’
5	Go Bananas!	Monthly parent meetings Activities with children Update notice board	Reflection and monitoring of goals (consented parents) Information sheets – Snack swaps, Snack and breakfast ideas, Make walks interesting Monthly challenges – The Banana Challenge, NO TV for 5 days, Family activity challenge, Our family achievement sheet, Our family reward chart	Ideas for no TV Activities with bananas End of programme preschool party – with fruit skewers The Banana Challenge cooking activity with parents Physical activities with parents Learning a new song ‘Buy me a banana’

Parents were also given monthly newsletters by preschool staff. These newsletters provided dietary and physical activity information and tips for home-based tasks to increase physical activity and healthy eating and decrease screen time. Active BCTs distributed via the newsletters were self-monitoring of behaviour, goal setting, action planning and coping planning.

Parents were asked to meet with preschool staff members once a month to set goals, monitor and review family health behaviour using written materials provided within the newsletters. The families were encouraged to share ideas, achievements, photographs and drawings of activities on a communal preschool information board. Home-based tasks included family cooking and tasting, ‘No TV day’ and increasing family ‘active time’ challenges. Preschool staff were given the choice of organising the monthly staff/parent meetings using an individual or group-based setting; this allowed each centre to tailor the mode of delivery according to preschool resources and parental preferences.

Preschool staff attended an intervention training session at the university or at their place of work and the intervention was delivered by preschool staff to all children. Information about the intervention format was provided to all families in the participating preschools and all families were encouraged to participate. However, 36 parents agreed to complete additional tasks (as described below) and for anthropometric measurements of their child to be taken. The demographic characteristics of these families are described in Table 3.

**Table 3:** Descriptive data of families participating in data collection

	Intervention schools *n* = 2	Wait schools *n* = 2	Total *n* = 4
Families (n)	15	21	36
Parent gender (n) M – F	3 – 12	4 – 17	7 – 29
Child gender (n) M – F	5 – 10	13 – 8	18 – 18
Child age (n) 3 years – 4 years	11 – 4	18 – 3	29 – 7
Child full time/part time n (%)	0 – 15 (0 – 100)	17 – 4 (81 – 19)	17 – 19 (47 – 53)
Parent education n (%)			
Some secondary school	1	3	4 (11)
Completed secondary school	6	5	11 (31)
Some additional training	5	8	13 (36)
Undergraduate education	2	1	3 (8)
Postgraduate education	1	3	4 (11)
Parent marital status n (%)			
Single	4	7	11 (31)
Married/cohabiting	11	14	25 (69)

### Primary outcome measures

The main aim of a feasibility study is to determine which data collection methods and tools would be acceptable to the targeted population and if trial and intervention procedures are acceptable and feasible [40, 41, 53].

Feasibility of trial procedures and acceptability of the proposed intervention was measured through the recruitment rates; completion rates of data collection tasks; lost to follow-up (parents); adherence to the proposed activities by preschools and parents; and number of pictures taken by children/families of meals consumed.

### Secondary outcome measures

#### Anthropometric data

##### Children

- Height and weight (Body Mass Index (BMI) percentiles, UK-WHO charts) [54]
  Each child had these measurements recorded on an individual data collection sheet. Using equipment purchased from Chasmors, London, UK, height was measured to 0.1 cm using a Leicester portable height measure with the head in the Frankfort plane, and weight to 0.1 kg using Tanita scales TBF-300MA (TANITA Corporation, Tokyo, Japan). Children remained clothed but were asked to remove shoes and objects from pockets. Body mass index (BMI) was calculated using the BMI percentiles from the UK-WHO charts.

#### Physical activity and eating behaviours

Data on physical activity, sedentary behaviour, eating, portion size and eating environment was collected for 4 days (two weekend days). These assessments are detailed below.

##### Children

- Physical activity was measured by either Actigraph GT1M accelerometer or ST-101 pedometer (two preschools were provided with accelerometers (*n* = 18) and the other two with pedometers (*n* = 14) due to lack of available accelerometers).
- Portion size and eating environment (home, school, restaurant, with family alone etc.) were assessed by photographs taken with a digital camera provided to children. Children were instructed to take pictures of all the meals and snacks consumed as this gave further contextual information to the food diary task - for more detail and results of this method see McSweeney et al., [55].

##### Parents

- Child eating behaviours: Completion of a 4 day food diary for their child; this was based on the Food Assessment in Schools Tool (FAST) [56]. The food diary is divided into four daily time slots and the parent was prompted to record all meals, snacks and drinks consumed by the child in these time periods.
- Sedentary behaviours: parents’ recorded the time in hours/minutes their child spent watching TV/DVDs in the daily diary. Family ‘active’ time (hours/minutes) was recorded in the diary by parents with instructions to record joint family behaviours such as walking, swimming etc.

##### Observational measures

- Food intake: child food intake when at preschool (2 week days) using a food checklist was recorded. Researchers itemised all foods eaten by child to be later entered into the FAST [56] diary.

### Process evaluation

Intervention feedback was an on-going process and was collected monthly in multiple forms: a) evaluation questionnaires collected from preschool practitioners: b) observations of intervention material actually used in the preschool; c) photographs of family and preschool activities (e.g., cooking challenges); d) completed parental worksheets (as described in Table 2), and; e) informal verbal communication with staff members (e.g., feedback of the activities and children and parent reactions), which was recorded in field notes.

At the end of the intervention, NPs were interviewed using a semi-structured interview and parents were asked to complete a questionnaire. Both the semi-structured interview as well as the questionnaire asked views on aspects of the intervention such as: recruitment and data collection methods; goal-setting activities; intervention materials and activities; how children interacted with intervention; and, whether they would do anything differently if the intervention was repeated.

### Analysis

A sample size of four schools is considered to be sufficient for a feasibility study, when a feasibility study is a small cluster randomised controlled trial, a power calculation is not normally undertaken [57]. The sample size should be adequate to estimate the critical parameters for example, the recruitment rate.

Participant recruitment, rejection, retention and attrition rates were tracked to assess how preschool centres and parents responded to being approached to join the study and also retention rates once they joined the study.

Measurements for weight, BMI percentiles, physical activity, sedentary behaviour, diet and questionnaire results were analysed using descriptive statistics for all participants. Completion of measures and questionnaires was recorded to assess adherence and feasibility for using these data collection methods with participants in future studies. Table 4 describes and summarises how each form of data collected was analysed, this included dietary analysis (the food diary), thematic (e.g., staff interviews) and visual content analysis (e.g., food photographs).

**Table 4:** Data collection tools and analysis summary

Data	Who?	Analysis
Food diary [42]	Parents Research staff	Dietary analysis: Food groups and nutrients by intervention/control groups
TV/DVD viewing diary	Parents	Comparison by intervention/control groups of hours engaged in sedentary (screening) activities
Family ‘active time’ diary	Parents	Thematic analysis
Child physical activity (accelerometer or step counter)	Children	MVPA and step count comparison by intervention/control groups
Food photographs record	Children/Parents	Visual content analysis
Anthropometric measures (height and weight)	Children	BMI comparison by intervention/control groups
Acceptability and feasibility of Information sheets (PA, diet and SB)	Parents/Staff	Feedback by questionnaire and/or interview (thematic analysis)
Acceptability and feasibility of Fruit/veg and cooking challenges	Children/Parents	Informal feedback from staff/parents and children Photographic evidence on notice boards Questionnaire and/or interview (thematic analysis)
No TV day challenges	Children/Parents	Informal feedback from staff/parents Questionnaire and/or interview (thematic analysis)
Goal-setting meetings and monitoring sheets	Parents/Staff	Informal feedback from staff/parents Questionnaire and/or interview (thematic analysis)
‘Sharing Tips’ sheets	Parents	Evidence of use on notice boards Questionnaire and/or interview (thematic analysis)
Shared notice board	Children/Parents/ Staff	Evidence of use (photos) Questionnaire and/or interview (thematic analysis)
Preschool-led activities	Children/Staff	Staff feedback by interview (thematic analysis)

## Results

### Recruitment

Of the 40 preschools contacted 4 consented to participate in the study. Within the 4 preschools, 36 families out of a possible 121 families (30%) consented to participate (*n* = 15: intervention, 21: control group, see Fig. 1). Table 3 describes the participant characteristics, including parental education level; which served as a proxy measure for social economic status (SES).

### Primary outcomes

#### Completion rates

Thirty-six parents in total agreed to take home a data pack which comprised of the 4-day diary, an accelerometer or pedometer and a digital camera for child use. At baseline 32 parents completed aspects of the data collection. Table 5 displays the parents’ adherence to data collection methods.

**Table 5:** Parent’s adherence with data collection tools

Outcome measure	T1 - Baseline	T2 – Follow up
n	32	26
Food diary %	81	58
TV diary %	78	58
Family activity diary %	41	38
PA monitors %	66	58

#### Lost to follow-up

At follow-up 27 families accepted a data pack, there was an increase in completion of all tasks (12 to 19%), however, there was also an increase in the number of families not completing any tasks. The Consolidated Standards of Reporting Trials (CONSORT) diagram in Fig. 1 illustrates the retention rates and numbers of participants available for analysis purposes.

#### Number of pictures taken of meals

The provision of digital cameras to record dietary intake, in order to include children in the data collection process and to give context to the food diaries proved successful with many families: 78% of children/families used this at baseline and 67% at follow-up. In total 273 photos were recorded, 204 (75%) of which were of food items. Non-food items included shots of household objects such as the TV, their parent’s bed and toys.

### Secondary outcome measures

#### Anthropometric measures

From the total sample 23% of children were classified as overweight or obese.

#### Physical activity

As previously discussed some of the children had their physical activity levels measured by an Actigraph accelerometer and some were provided with pedometers. Accelometers report levels of moderate to vigorous activity (MVPA); pedometers the number of steps taken per day. A daily step count of 13,874 equates to 1 h MVPA in children [58]. Eleven out of 14 (78%) children provided pedometer data and 11 out of 18 children (61%), accelometer data. On average all the children failed to reach the recommended 180 min of MVPA per day.

#### Sedentary behaviour

Seventy-eight percent of parents completed the TV section of the diary. The children’s TV/DVD viewing habits were used as a proxy measure for sedentary behaviours. The length of viewing times ranged from 42 min per day to over 7 h per day. However, some parents did report that the TV was left on all day and the child may have been doing other activities at the same time. On average the children in all schools watched more than 3 h of TV per day.

#### Dietary measures

Eighty-one percent of parents completed the FAST food diary. Some key findings included: both groups of children consuming an average of 33% fat of total energy. The intervention group consumed 13% non-milk extrinsic sugars (NMES) of total energy and the control group 18% of total energy. Other nutrients were also similar between the groups with the exception of Vitamin C with the intervention group consuming 71 mg and the delayed group consuming 84 mg.

#### Family active time

Forty-one percent of parents completed the family active section of the diaries; parents included entries such as: ‘[name of child] walked with dad to nursery (20 min)’ and ‘Whole family went to swimming pool (1 h)’.

#### Observational measures

Whilst at preschool, the children’s dietary intake was observed and recorded by the researcher over the 2 week-day data collection period. In all of the preschool centres the children’s snack consumption was observed and recorded in the child’s FAST diary. In the cases where the parent forgot to bring the FAST diary to preschool with the child, notes were written and the researcher added the information to the diary at a later date. In two of the centres some children were cared for over the lunch-time period, therefore, school lunch or home-made packed lunch was observed and recorded also. Inevitably, other aspects of the preschool routine were observed. In one centre, staff were observed eating cakes and chocolate whilst children were given a fruit snack. Furthermore, two centres provided children with weekly rewards of chocolate for ‘good attendance’ and ‘good behaviour’.

### Process evaluation

On-going evaluation throughout the intervention took place in the form of: a) monthly feedback forms completed by the teachers; b) photographic evidence by the children/parents (information boards); c) evidence of intervention materials used; d) evidence of activity uptake by the families (i.e., goal-setting and monitoring sheets completed by parents), and; e) informal communications and observations by the researcher with NP’s.

#### Staff feedback

Most communication and interaction during the intervention period was with the preschool teachers. It was difficult to determine how much the nursery practitioners were engaging with the project. Two preschool teachers one from each of the intervention schools were interviewed.

There was consensus that the recruitment and engagement of parents in all types of preschool events and activities was an on-going challenge and one teacher felt that more parents might have volunteered to participate in the intervention with an additional information meeting before the parent recruitment day,

‘We felt that if we’d made it more of a joint meeting between ourselves and invited the parents in, made sure all the parents came in and were fully aware of what was going to happen, so have the staff’s involvement as well and saying that we’re going to have these meetings and can you come and make sure that you sign up, that you are willing to come to these meetings’ (Teacher intervention centre1).

Teachers’ reported observed changes both in parents and children: raised parental awareness of the importance of healthy lifestyles at an early age, families doing more activities together and the children talking about staying healthy:

‘They’ve [the children] been talking a lot more about keeping healthy and what’s healthy and what’s not healthy, so definitely must have been talking about it at home as well as here’ (Teacher intervention centre 2).

It was evident from all forms of feedback, including photographic and verbal, that the practical tasks such as the cooking challenges engaged the families in both preschools in a significant manner. The teachers reported this as a strength of the intervention:

‘It’s been really lovely to see in the children that they’ve been so excited bringing and showing what they’ve done and so it means that it has had a real impact. They’ve [the parents] gone home and they’ve done things with their children that I don’t necessarily think they would have done at all and that’s important’ (Teacher intervention centre 2).

#### Parental feedback

Thirty-eight percent of parents returned questionnaires. The reasons presented for lack of meeting attendance were mostly rooted with work commitments. However, despite a reported low level of attendance to the goal setting discussion meetings (formal attendance figures were not collected), 62% of parents’ demonstrated evidence of setting goals for their family at home. Moreover, the majority of parents, at intervention end, (either by the questionnaire or the returning of goal-setting and monitoring sheets) indicated that some positive health behaviour changes within the family setting had been made:

‘We watched less TV and took part in more physical activities’ (Parent intervention centre 1).

In accordance with the staff feedback, the most positive comments from parents related to the cooking challenges:

‘It was funny and enjoyable…we enjoyed creating the new recipes’ (Parent intervention centre 2).

## Discussion

### Recruitment - preschools

Recruitment of preschool centres was time-consuming as administrative ‘gatekeepers’ had to be negotiated via several telephone calls before access to the head-teacher was granted. Consent for the preschool to participate was granted by a senior member of staff such as the head-teacher or assistant head-teacher.

### Staff members

The level of consultation which took place between senior staff and nursery practitioners in relation to participation in the intervention is not known. Some staff members appeared uncertain about their involvement and what was expected of them. They tended to avoid corresponding with the parents about the intervention and left all communication to the class teacher and/or researcher, especially during the parent recruitment sessions. The findings from this study corroborates other studies in that despite having commitment to a project from ‘gatekeepers’ such as head teachers, this does not necessarily transfer to those who are working at ground level [59, 60]. It is not sufficient to have the permission of ‘gatekeepers’ to implement a study but all members of staff need to feel included in the development and potential deployment of an intervention [59] if this is to be successful. An example of this is highlighted in a study with preschool staff in Norway [61]. The Norwegian staff expressed some feelings of unease with the intervention and it was concluded that the staff needed more groundwork preparation and an indication of potential gains in participating. The teachers in the SKIP study were active and enthusiastic, however, it was challenging trying to engage with the nursery practitioners. Moreover, the nursery practitioners were reluctant to attend the training session out-with work hours. Recent work by De Silva et al., [62] in Australia highlights the importance of providing preschool staff with support to develop and implement policies. Furthermore, positive and consistent reinforcement for preschool practitioners during an intervention may enhance morale and increase the likelihood of adherence [63]. Finally, if all staff are to participate in the delivery of the intervention, protected time needs to be allocated for their training needs.

### Parents

Better practitioner engagement with the intervention may have transferred to the parents. It was difficult to know for certain why the parental recruitment rate (30%) was so low. Recruiting participants to engage in a research project is considered to be the most challenging aspect of the research process [64]. As identified in Social Marketing literature, it is important that potential participants in an intervention have a ‘reason to care’ and feel ready for change [65]. Thinking about the refinement of the procedures we conclude that an initial joint meeting involving research staff, all preschool staff members and parents may remove some of the uncertainty and mystery of participation. In addition, engaging parents/families in relevant ‘fun’ activities, such as the cooking activities, prior to recruitment may be beneficial and a fun way to introduce them to the study. Many parents who declined to sign-up to the SKIP study cited lack of time; however, if the intervention/topic ‘spoke’ to them, this possibly would not have been a deterrent.

As a result of the low recruitment rate for this study a sample size calculation for a potential main trial cannot be made. What we have learned from this feasibility and acceptability study will allow us to refine trial and intervention procedures. With this refined protocol the aim is to run a pilot study with at least 35 participants in each group [66]. The barriers and potential problem-solving strategies we have highlighted following the analysis of the data from this study will assist with recruitment to a future pilot trial.

### Primary outcomes

Parental compliance with the data collection tasks was mixed; the parents were most likely to complete the food and TV diaries. One mother commented that she kept the camera and physical activity monitor out of her children’s reach (she had 5 children) for fear of them being damaged. At follow-up there was an increase in the number of families not completing any tasks, parents gave no other specific reasons for failing to complete the baseline measurements; therefore it was difficult to determine why the repeat measurements induced further levels of attrition. It simply may have been a decline in the novelty factor of taking part. The use of digital cameras to record dietary intake proved successful with many of the families and has previously been trialled in several studies with older children [67–70]. This study demonstrates that photography may be a tool suitable for use in younger children also [55].

### Secondary outcomes

The findings from the secondary outcome measures were consistent with other studies. Twenty-three per cent of the sample were classified as overweight or obese. The national percentage of overweight and obesity in reception age children (4/5 year-olds) from the 2010/11 National Childhood Measurement Programme (NCMP) data was 22.6% [71]. Not only is there evidence that childhood overweight and obesity can be linked with numerous long-term and immediate health risks [72] but there is also an association between overweight and a lower self-concept (how one perceives themselves) in girls as young as 5 years [73]. Therefore, preschool aged children may already be at increased risk of negative psychological impacts of obesity [73, 74].

On average all the children failed to reach the recommended 180 min of MVPA per day. This was in line with other studies which have reported very low levels of physical activity in preschool children [75–77]. Previous studies suggest that preschool settings may have great scope for improving physical activity [27, 28]. Parents of young children have reported that although they feel responsible for their child’s diet, they were less decisive about PA and relied on school/preschool staff to ensure their child was adequately active [30, 78]. The 2009 NICE guidelines for promoting physical activity for children and young people, recommend that families should be given physical activity ‘homework activities’ which children and parents/carers can do together [79].

It was reported that on average all the children watched more than 3 h of TV a day. This finding is of concern as it has been reported that children who watch more than 2 h per day of TV consume less fruit and vegetables, more high energy drinks [80] and have a higher consumption of calorie rich ‘unhealthy’ food [81–83]. However, as BMI and TV viewing may not be associated until the child is 7 years of age [81]; this emphasises the importance of reducing sedentary behaviours in younger children. Although the impact on weight status may not be apparent until they are older, the learned behaviours can become habitual [84]. Therefore, it is of great importance that parents and NPs are made aware of the current viewing habits of young children and the possible long-term impact on their health.

The nutrient intake results from the children were comparable with findings from the National Diet and Nutrition Survey (NDNS); suggesting the FAST diary method [56] was a suitable tool for collecting preschool children’s dietary data. As with the NDNS results, children presented higher intakes of saturated fat and non-milk extrinsic sugars than daily recommendations. However, children in the current study recorded lower levels of Vitamin A, Vitamin D, non-starch polysaccharides (NSP) and sodium than other surveys.

### Observational measures

Findings from the present study highlighted the apparent difficulty or preschool intentional lack of compliance with preschool health policies such as allowing high energy dense snacks and foods. Moreover, some practitioners appeared to be unaware of the impact of their own consumption behaviour on children (i.e., modelling). However, some practitioners did voice feeling ‘guilt’ in giving the children ‘treats’ when the researcher was observing these behaviours. Moreover, in the primary qualitative study [45] it was evident that the provision of ‘treats’ and birthday cakes brought into preschools from home was a regular occurrence. As discussed previously, there is a paucity of interventions targeting children in preschool settings in the UK. These findings highlight the need to further explore practitioners’ personal perceptions of health and how the embedding of food policies which promote healthier food being brought into the preschool setting from home can be implemented [85].

### Process evaluation

Although the preschools were given the same training, information and tasks to complete, it was apparent that each school tailored the implementation to suit their own setting. This is significant finding [86], as it is essential that the intervention can function in a ‘real’ world situation and is capable of being integrated into the daily routine [35]. However, it was reported that there was some difficulty with completing all the intervention tasks due to curriculum demands and obligation to comply with primary school demands (all the preschools were based within a primary school setting). One teacher suggested that future preschools participating in such an intervention may want to plan the intervention into the curriculum in advance. This would not only benefit the implementation of the intervention but may also provide staff with involvement and ownership of the project. This could coincide with In Service Training (INSET) days and contribute to staff professional development. Potentially, a health officer working with several schools could be employed to support staff and intervention implementation.

The teachers displayed enthusiasm for and adherence with the intervention. The materials and activities were liked and reported as suitable. It was reported that a significant strength of the intervention was the capacity to encourage parents to become involved with their children, especially in the practical home tasks.

The teachers reported parental difficulties with the goal-setting meetings and activities, however, findings suggest it was not the goal-setting tasks per se that parents were opposed to, but the actual meeting and discussing of the goals with staff members. The parents may have found discussing family behaviours with the teacher too personal or overly intrusive fearing judgement [87]. Previous US work reports that parents of a lower SES may have greater concerns about privacy [88]. Moreover, the parents may have questioned the teacher’s qualifications to discuss such matters. Results from the primary qualitative study [45] found that parents were worried about ‘being told what to do’ and being thought of as ‘bad parents’. This highlights the importance of exploring other methods of feedback such as email, or an on-line website. Results from a telephone survey conducted in Australia in 2010, suggested that when trying to engage parents in interventions to prevent obesity in childhood, support services such as personalised mail/email support would be the most acceptable method to parents [89]. In addition, further strategies for engaging NP engagement and interest would be beneficial [63].

The most successful and accepted elements of the intervention were the family cooking challenges and the shared preschool photograph/notice boards. The ability of these tasks to engage the parents is significant and should perhaps form the basis of future preschool behaviour change interventions. There was also evidence of parents attempting other intervention tasks such as increasing ‘family active time’, the ‘No TV challenges’ and trying new recipes. Therefore the findings indicate an acceptability of the methods and materials by the parents; however, this was demonstrated mostly by the ongoing informal observations and collected task sheets as very few parents returned the intervention end evaluation questionnaires. It proved frustrating knowing that many parents had engaged with the intervention but felt unable to provide feedback.

### Intervention refinement

The following proposals for the refinement of the intervention for future implementation are recommended. The researcher, preschool staff and parents should meet for a pre-intervention meeting to collaboratively discuss the intervention purpose, procedures and importance of data collection methods. Engaging parents in relevant ‘fun’ activities prior to recruitment may encourage more parents to participate. The parental self- monitoring goal implementation and activity sheets should be simplified for optimal understanding and to potentially increase completion. In addition, ways to systematically collect process measures data, for example the parent-completed activity and feedback forms, should be identified. Preschool staff should be issued with more explicit physical activity and playground games ideas to improve use of provided activity equipment. Recipes should be provided with the cooking challenge activities, as it was highlighted by preschool staff that not all families were familiar with the provided fruits and vegetables. The delivering of a refined, delayed intervention provides an incentive for the wait list control preschools to participate, because instead of just providing control data, they later receive a refined intervention [43].

## Strengths and limitations

Several strengths of this work can be reported. The study was able to demonstrate the acceptability of the intervention tasks and materials and it was evident that families were engaging with aspects of the intervention such as the cooking challenges. The intervention was underpinned by two theoretical models which aided the development of and implementation of the intervention and adds to the body of evidence.

The recruitment rates were very low; this was a doctorate study and time and resources limited further recruitment measures. However, those parents that did take part were from a varied SES thus it seems it is possible to reach a wide range of the population. Parental education level was used as a proxy measure for social economic status (SES) and it is acknowledged that a single proxy measure is unlikely to provide accurate information; ideally multiple proxy measures would be measured [90]. We did not have the characteristics of those parents not taking part. Therefore, the sample may not necessarily be representative of the whole school; if this was a full trial and not a feasibility study, this could be regarded as a limitation. The lack of opportunity to meet with all staff members may have had a negative impact on parental recruitment and staff engagement. Ideally, more time would have been allocated to the staff training; 1 h of training for the complexity of the intervention was insufficient. However, time was a rare commodity for the NPs; especially those who were not willing to attend sessions out-with work hours.

## Conclusions

The preschool years have been highlighted as a critical period for obesity prevention [22] and interventions which target strategies and techniques which aid parents in modifying their child’s diet and physical activity patterns [34] have been recommended.

Despite some issues with staff engagement and parental recruitment, the preschool teachers reported that certain elements of the intervention, such as the cooking challenges and shared notice boards, were successful in engaging families with preschool age children. In future studies, it may be beneficial to engage families in ‘fun’ activities, such as the cooking challenges before any formal recruitment is attempted. Furthermore, large scale studies are needed to further explore the suitability and effectiveness of the role of nursery settings for childhood obesity prevention.

It was found that the practices of some preschool settings and practitioners contradicted reported preschool health policies. The findings highlight the need to not only focus on family health behaviours but to address the whole ‘obesogenic’ environment to which a child is exposed. The findings from this study will contribute to the small pool of UK research in understanding which strategies for the prevention of obesity in preschool children are most acceptable to preschool staff and parents. It is hoped that expansion of research in this area will contribute to the government aim of reducing childhood obesity to 2000 levels by 2020.

## Abbreviations

BCT: Behaviour change techniques
BMI: Body mass index
CONSORT: Consolidated standards of reporting trials
EYFS: Early years foundation stage
FAST: Food in schools tool
INSET: In service training
MVPA: Moderate to vigorous physical activity
NCMP: National Childhood Measurement Programme
NDNS: National Diet and Nutrition Survey
NP: Nursery practitioner
NSP: Non-starch polysaccharides
OC: Operant conditioning
PSA: Public service agreement
SCT: Social cognitive theory
SES: Social economic status
UK: United Kingdom

## Footnotes

1 The manual and materials from the programme are available from the corresponding author.
